# A stochastic model explains the periodicity phenomenon of influenza on network

**DOI:** 10.1038/s41598-021-00260-3

**Published:** 2021-10-25

**Authors:** Hong Yang, Zhen Jin

**Affiliations:** 1 Complex System Research Center, Shanxi University, Taiyuan, 030006 Shanxi China; 2 Shanxi Key Laboratory of Mathematical Techniques and Big Data Analysis on Disease Control and Prevention, Shanxi University, Taiyuan, 030006 Shanxi China

**Keywords:** Diseases, Mathematics and computing

## Abstract

Influenza is an infectious disease with obvious periodic changes over time. It is of great practical significance to explore the non-environment-related factors that cause this regularity for influenza control and individual protection. In this paper, based on the randomness of population number and the heterogeneity of population contact, we have established a stochastic infectious disease model about influenza based on the degree of the network, and obtained the power spectral density function by using the van Kampen expansion method of the master equation. The relevant parameters are obtained by fitting the influenza data of sentinel hospitals. The results of the numerical analysis show that: (1) for the infected, the infection period of patients who go to the sentinel hospitals is particularly different from the others who do not; (2) for all the infected, there is an obvious nonlinear relationship between their infection period and the visiting rate of the influenza sentinel hospitals, the infection rate and the degree. Among them, only the infection period of patients who do not go to the sentinel hospitals decreased monotonously with the infection rate (increased monotonously with the visiting rate), while the rest had a non-monotonic relationship.

## Introduction

Influenza is an respiratory infection caused by a virus. Since the outbreak of the Spanish flu in 1918, there has been a large amount of literature on influenza, among which mathematical model is a very important tool for studying influenza^1–4^. The traditional deterministic mean-field model^5–8^ regards different populations as uniformly mixed and ignores the differences in the contact process and behavior among individuals. In general, this contact process can be viewed as a network in which nodes represent individuals and edges represent contacts between individuals. Network has its unique topology structure, such as degree *k* (i.e. the number of edges connecting to one node), degree distribution (i.e. *P*(*k*) denotes the probability that a randomly chosen node in the network has degree *k*), clustering coefficient (i.e. the average probability that two neighbors of a node are themselves neighbors) and so on^9,10^. The topological structure of complex network may lead to some results of overthrowing the traditional, for instance, there is no epidemic threshold in a scale-free network which follows a degree distribution of power-law ($P(k)\propto k^{-\gamma }$ when $1<\gamma \le 3$), for the SIS model^11^ raised by Pastor. So far, there are many epidemic disease modelling methods based on different features of the network, such as pairwise model^12^, edge-based compartmental modelling^13^, degree-based modelling^14^ and effective degree modelling^15^.

The recurrent of epidemic brings a big challenge for people to treat the disease^16^. Many diseases exhibit the phenomenon of annual, biennial, multi-annual and irregular oscillations, such as measles, whooping cough, influenza and so on. The prediction of these diseases by using deterministic dynamic model satisfactorily both the internal mechanism and persistence properties exhibited by case report remains elusive, especially the multi-year periodicity phenomenon, which can be regarded as the comprehensive results of nonlinear dynamics, random factors and seasonal forcing^17^. Many works of literature related to seasonal cases using the deterministic dynamic model where the contact rate is temporal^18,19^, and the period is artificial, yet, the endogenous period is not always fixed at 1 year in reality. By using some methods, such as Fourier transform and wavelet analysis, which are used in the following part, we can get the endogenous frequency of these case data, which better conforms to the actual situation and helpful in controlling a pandemic.

In addition, noise is usually considered a nuisance, yet it is unavoidable in reality. However, many researches show that it may play a constructive role. Noise interacting with the deterministic dynamic system may lead to some different results: (1) extinction^20^, the disease may die out even though the basic reproduction number $R_{0}$ is larger than one in the deterministic system; (2) fluctuation^21^, the autonomous system is allowed to remain oscillation around the unique nonzero stable equilibrium, which is one of the main themes of this paper; (3) stochastic phase transition^22^, the system is caused to shift between different attractors.

Distinction from the general Susceptible-Infectious-Recovered-Susceptible model for influenza, which is abbreviated as *SIRS*, we divide the infected people into two part: ones who go to the sentinel hospitals (*H*) and the others (*I*) who do not. In this paper, we consider the *SHIRS* model based on the degree to describe analytically the fluctuations produced by demographic stochasticity, in which, the method called van Kampen’s system-size expansion^23^ is used. The power spectral densities (PSD) of the number of infected and susceptible individuals can be derived to describe the effect of stochastic amplification and heterogeneity by using this method, which based on individual-based formulation has been applied to epidemiology^24,25^. However, the researches which involve in the relevant work on network are still relatively few.

**Figure 1:**
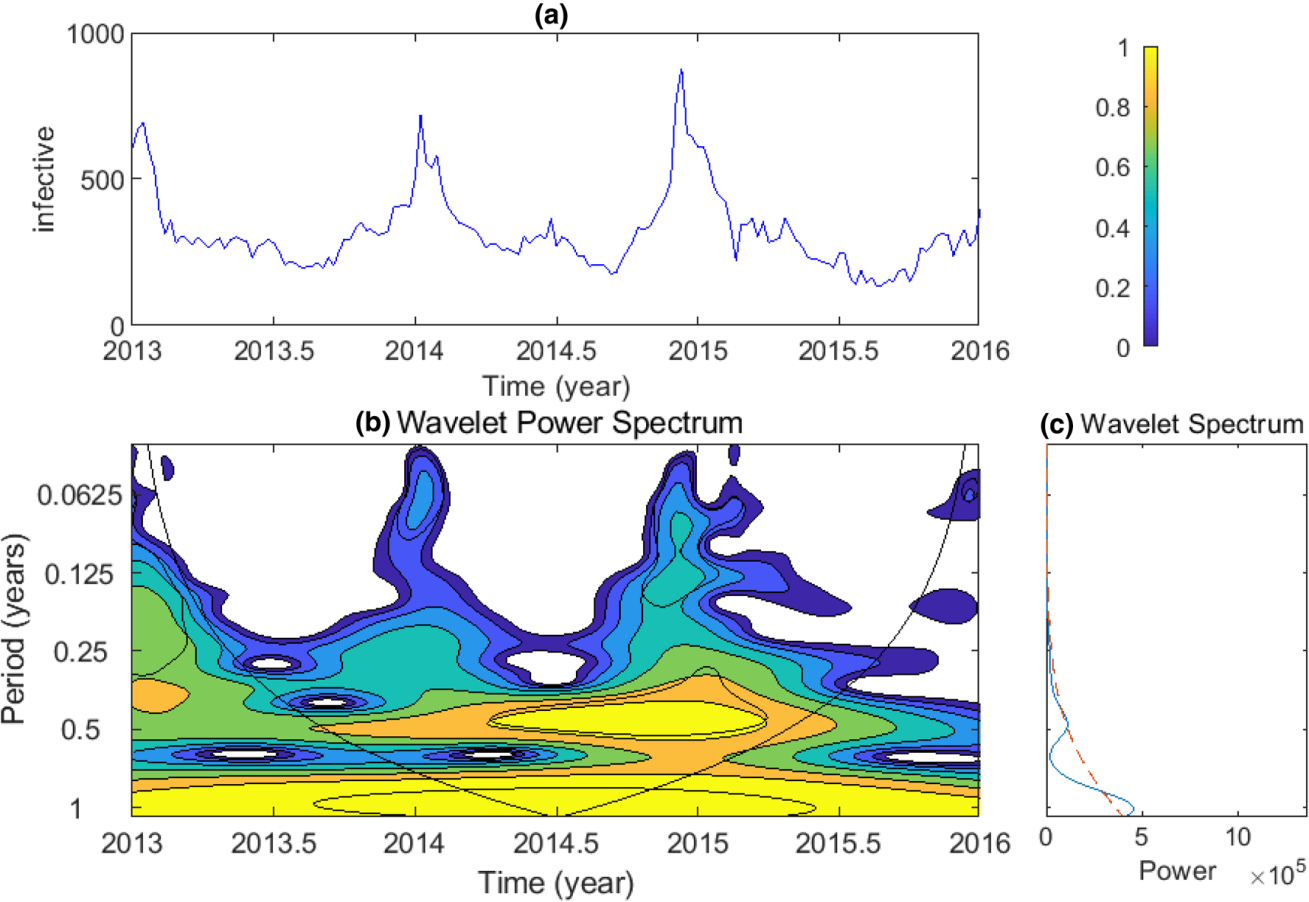
Temporal periodicity analysis of influenza in Taiyuan using the wavelet method. (**a**) Weekly infective number of influenza from 2013 to 2016 of Taiyuan sentinel hospitals. (**b**) The wavelet spectrum analysis corresponds to time series of the data of (**a**). High power values are colored in yellow, orange and cyan denote intermediate power, blue denotes low one. Note the black line is the $95\%$ confidence level. Only patterns within these lines which can neglect the edge effects are considered reliable. (**c**) The average wavelet spectrum (blue line) and the corresponding $95\%$ confidence contour(red).

The influenza week data from the sentinel hospitals of Taiyuan since 2013–2016 have a period of semi-annual to annual through wavelet method^26,27^ illustrated by Fig. 1. To investigate the relationship between the multi-span phenomenon of period and network structure, we first develop a fully stochastic heterogeneous *SHIRS* model. Then, the PSD is obtained from the Langevin equations by Fourier transformation which provides a prediction for the dominant period of the flu, and the sensitivity of parameters is analyzed, in which the values of the corresponding parameters are obtained from the part of data mentioned above by using least square estimation. Finally, we discuss the limitations and extensions of this method. Our analysis sheds new light on the importance of heterogeneity for influenza outbreaks and persistence.

## The stochastic SHIRS model on network

### Stochastic modelling

In this paper, we divide the population *N* into four classes, in which *S* denotes the number of individuals who are susceptible to disease, but are not yet infected at the moment; *H* or *I* represents the size of those who are infected with the disease and are able to transmit disease to the susceptible people by contacting with them, the only difference between them is whether to go to the sentinel hospitals, in which *H* denotes the infected people who have gone to the sentinel hospitals, yet *I* not; *R* is the size of people who have recovered from the infected and immune to the disease.

The number of contacts (i.e. the degree) is extraordinary different because of the diversity of factors between people like age, profession and so on. For example: people in school due to the factors of enormous population density has a higher degree. So degree is a important indicator to depict the heterogeneity. Similar to the modelling method proposed by Pastor^11,14^, we mark off the whole population into *n* groups according to the degree, i.e. $N=N_{1}+...+N_{n}$, in which, $N_{k}$ is the size of population with degree $k, (k=1,2,...,n)$. $P(k)=\frac{N_{k}}{N}$ is the degree distribution of the network, $\langle k\rangle$ is the mean degree, i.e. $\langle k\rangle =\sum \limits _{k=1}^{n}kP(k)$, similarly, $\langle k^{2}\rangle =\sum \limits _{k=1}^{n}k^{2}P(k)$ is the secondary moment of the degree distribution.

Based on the classification of disease we mentioned above, people in one group can be divided to four classes: $S_{k}, H_{k}, I_{k}, R_{k}$, in which $S_{k}$ denotes the number of the susceptible people with degree *k*, and $H_{k}, I_{k}, R_{k}$ have the similar meanings. We assume the total population size and degree distribution do not change with time, which implies that the $N_{k}$ is a constant. By using the relationship expression: $R_{k}=N_{k}-S_{k}-H_{k}-I_{k}$, we can obtain the recovered individual $R_{k}$ under the new $(S_{k},H_{k},I_{k})$ framework, so the state of the system can be defined by the three integers $(S_{k},H_{k},I_{k})$.

Though the deterministic ordinary differential equation can be viewed as the approximate solution of the stochastic differential equation when the number of population is large, the stochastic modelling method is more accurate, in which continuous time Markov modelling is used in our paper.

**Figure 2:**
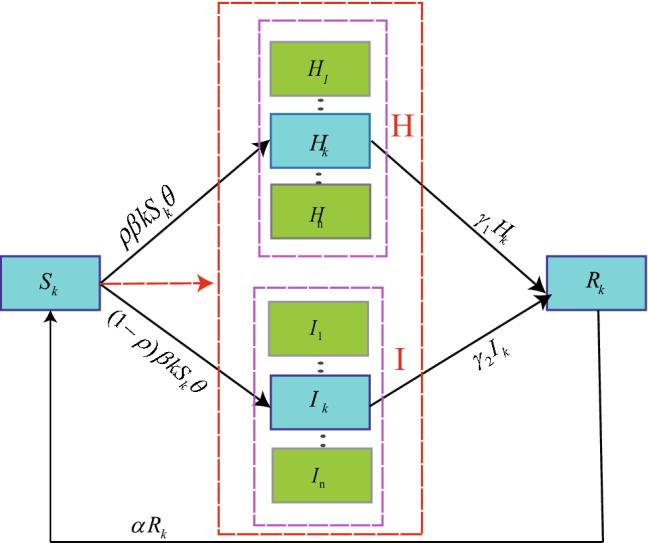
Schematic diagram of the *SHIRS* based on degree, the parameters meaning are listed in Table 2.

At first, we should understand the whole transfer processes of the disease and the corresponding transfer rate, which has been listed in Table 1. Figure 2 has shown the schematic diagram of our model, from which we can know people in one compartment may enter into another due to the three processes:

**Table 1:** List of transition rates.

Event	Transition	Rate
Infection	$S_{k}\rightarrow S_{k}-1$, $H_{k}\rightarrow H_{k}+1$	$\rho \beta kS_{k}\theta$
	$S_{k}\rightarrow S_{k}-1$, $I_{k}\rightarrow I_{k}+1$	$(1-\rho )\beta kS_{k}\theta$
Recovery	$H_{k}\rightarrow H_{k}-1$	$\gamma _{1}H_{k}$
	$I_{k}\rightarrow I_{k}-1$	$\gamma _{2}I_{k}$
Lose immunity	$S_{k}\rightarrow S_{k}+1$	$\alpha (N_{k}-S_{k}-H_{k}-I_{k})$

#### Infection

$S_{k}$ may become infected by contacting with a infected individual in any group. i.e. $S_{k}{\mathop {\rightarrow }\limits ^{\rho \beta kS_{k}\theta }}H_{k}$, $S_{k}{\mathop {\rightarrow }\limits ^{(1-\rho )\beta kS_{k}\theta }}I_{k}$, where $\rho$ is the proportion of infected people who have go to the sentinel hospitals, $\beta$ is the probability of infection per contact, if the correlation of the states of the diseases and the degree have been neglected, under this condition, $\theta$ which means the probability of any chosen edge being connected to infected has the following form:

1 $$\begin{aligned} \theta _{A} \triangleq \frac{\sum \limits _{m=1}^{n}mH_{m}}{\sum \limits _{m=1}^{n}mN_{m}}, \theta _{I} \triangleq \frac{\sum \limits _{m=1}^{n}mI_{m}}{\sum \limits _{m=1}^{n}mN_{m}}, \theta \triangleq \theta _{H}+\theta _{I}, \end{aligned}$$

Because of the Markov property, the current system state just depends on the one of last time, so we use $T(\delta '|\delta )$ to denote the transfer rate of the system state changing from $\delta$ to $\delta '$, then the transition rate of the infection process can be written as:

2 $$\begin{aligned} T(S_{k}-1,H_{k}+1,I_{k}|S_{k},H_{k},I_{k})&= \rho \beta kS_{k}\theta ,\\ T(S_{k}-1,H_{k},I_{k}+1|S_{k},H_{k},I_{k})&= (1-\rho )\beta kS_{k}\theta . \end{aligned}$$

#### Recovery

Infective people can move out this compartment because of recovery over time, $H_{k}{\mathop {\rightarrow }\limits ^{\gamma _{1}}}R_{k}, I_{k}{\mathop {\rightarrow }\limits ^{\gamma _{2}}}R_{k}$, where $\gamma _{1}$ and $\gamma _{2}$ are recovery rates. This transition rate can be denoted as:

3 $$\begin{array}{l} T({S_k},{H_k} - 1,{I_k}|{S_k},{H_k},{I_k}) = {\gamma _1}{H_k},\\ T({S_k},{H_k},{I_k} - 1|{S_k},{H_k},{I_k}) = {\gamma _2}{I_k}. \end{array}$$

#### Lose immunity

The recovered people will lose immunity after some time, $R_{k}{\mathop {\rightarrow }\limits ^{\alpha }}S_{k}$, in which, $\alpha$ is the rate of lose immunity. The corresponding transition rate of this process is:

4 $$\begin{aligned} T(S_{k}+1,H_{k},I_{k}|S_{k},H_{k},I_{k}) = \alpha (N_{k}-S_{k}-H_{k}-I_{k}). \end{aligned}$$

Now, this continuous time Markov process can be modeled using a master equation, which has the general form:

5 $$\begin{aligned} \frac{dp(\delta ,t)}{dt}=\sum _{\delta '\ne \delta }T(\delta |\delta ')p(\delta ',t) -\sum _{\delta '\ne \delta }T(\delta '|\delta )p(\delta ,t). \end{aligned}$$

This equation describes the process of state transition of the system: some system states shift from $\delta '$ to $\delta$, meanwhile $\delta$ changes to the other system state $\delta '$, $p(\delta ,t)$ is the probability of the system state in the $\delta$ at time *t*. The explicit form of the master equation involving the three processes is given below:

6 $$\begin{aligned} \frac{d p(S_{k},H_{k},I_{k},t)}{dt}&=T(S_{k},H_{k},I_{k}|S_{k}+1,H_{k}-1,I_{k})p(S_{k}+1,H_{k}-1,I_{k},t)\\&\quad +T(S_{k},H_{k},I_{k}|S_{k}+1,H_{k},I_{k}-1)p(S_{k}+1,H_{k},I_{k}-1,t)\\&\quad +T(S_{k},H_{k},I_{k}|S_{k},H_{k}+1,I_{k})p(S_{k},H_{k}+1,I_{k},t)\\&\quad +T(S_{k},H_{k},I_{k}|S_{k},H_{k},I_{k}+1)p(S_{k},H_{k},I_{k}+1,t)\\&\quad +T(S_{k},H_{k},I_{k}|S_{k}-1,H_{k},I_{k})p(S_{k}-1,H_{k},I_{k},t)\\&\quad -[T(S_{k}-1,H_{k}+1,I_{k}|S_{k},H_{k},I_{k})+T(S_{k},H_{k},I_{k}-1|S_{k},H_{k},I_{k})\\&\quad +T(S_{k},H_{k}-1,I_{k}|S_{k},H_{k},I_{k})+T(S_{k}-1,H_{k},I_{k}+1|S_{k},H_{k},I_{k})\\&\quad +T(S_{k}+1,H_{k},I_{k}|S_{k},H_{k},I_{k})]p(S_{k},H_{k},I_{k},t). \end{aligned}$$

### The deterministic limit system

From the last section, the master equation has been obtained, which contains all information of the system, in this section, we will study the deterministic limit system corresponding to the (6).

For the mean values

$$\begin{aligned} \langle {S}_{k}\rangle&=\sum \limits _{S_{k},H_{k},I_{k}} S_{k}p(S_{k},H_{k},I_{k},t), \langle {H}_{k}\rangle =\sum \limits _{S_{k},H_{k},I_{k}} H_{k}p(S_{k},H_{k},I_{k},t),\\ \langle {I}_{k}\rangle&=\sum \limits _{S_{k},H_{k},I_{k}} I_{k}p(S_{k},H_{k},I_{k},t) \end{aligned}$$

may be obtained by multiplying Eq. (6) by $S_{k},H_{k},I_{k}$ in turn, and then summing over all the states of the system, the mean field theory takes the explicit form:

7a $$\begin{aligned} \frac{d\langle {S}_{k}\rangle }{dt}=\sum _{S_{k},H_{k},I_{k}}\{&- T(S_{k}-1,H_{k},I_{k}+1|S_{k},H_{k},I_{k})p(S_{k},H_{k},I_{k},t) \nonumber \\&-T(S_{k}-1,H_{k}+1,I_{k}|S_{k},H_{k},I_{k})p(S_{k},H_{k},I_{k},t) \nonumber \\&+T(S_{k}+1,H_{k},I_{k}|S_{k},H_{k},I_{k})p(S_{k},H_{k},I_{k},t)\}, \end{aligned}$$

7b $$\begin{aligned} \frac{d\langle {H}_{k}\rangle }{dt}=\sum _{S_{k},H_{k},I_{k}}\{&T(S_{k}-1,H_{k}+1,I_{k}|S_{k},H_{k},I_{k})p(S_{k},H_{k},I_{k},t) \nonumber \\&-T(S_{k},H_{k}-1,I_{k}|S_{k},H_{k},I_{k})p(S_{k},H_{k},I_{k},t)\}, \end{aligned}$$

7c $$\begin{aligned} \frac{d\langle {I}_{k}\rangle }{dt}=\sum _{S_{k},H_{k},I_{k}}\{&T(S_{k}-1,H_{k},I_{k}+1|S_{k},H_{k},I_{k})p(S_{k},H_{k},I_{k},t)\nonumber \\&-T(S_{k},H_{k},I_{k}-1|S_{k},H_{k},I_{k})p(S_{k},H_{k},I_{k},t)\}. \end{aligned}$$

When we substitute these transition rates of Eqs. (2)–(4) into the above and introduce the corresponding density variables in the limit $N_{k}\rightarrow \infty$,

8 $$\begin{aligned} s_{k}=\lim _{N_{k}\rightarrow \infty }\frac{\langle {S}_{k}\rangle }{N_{k}}, h_{k}=\lim _{N_{k}\rightarrow \infty }\frac{\langle {H}_{k}\rangle }{N_{k}}, i_{k}=\lim _{N_{k}\rightarrow \infty }\frac{\langle {I}_{k}\rangle }{N_{k}}. \end{aligned}$$

The corresponding deterministic equations of $s_{k}$, $h_{k}$ and $i_{k}$ can be obtained:

9 $$\begin{aligned} \frac{d s_{k}}{dt}=&-\beta ks_{k}\theta +\alpha (1-s_{k}-h_{k}-i_{k})=f_{1}(s_{k},h_{k},i_{k}),\\ \frac{d h_{k}}{dt}=&\rho \beta ks_{k}\theta -\gamma _{1}h_{k}=f_{2}(s_{k},h_{k},i_{k}),\\ \frac{d i_{k}}{dt}=&(1-\rho )\beta ks_{k}\theta -\gamma _{2}i_{k}=f_{3}(s_{k},h_{k},i_{k}), \end{aligned}$$

in which $\theta$ has the following equivalent form:

10 $$\begin{aligned} \theta _{h}=\theta _{H}=\sum \limits _{m=1}^{n}\frac{mP(m)h_{m}}{\langle k\rangle }, \theta _{i}=\theta _{I}=\sum \limits _{m=1}^{n}\frac{mP(m)i_{m}}{\langle k\rangle }, \theta =\theta _{h}+\theta _{i}. \end{aligned}$$

It is easy to obtain that for the system (9), the basic reproduction number $R_{0}=[\frac{\rho \beta }{\gamma _{1}}+\frac{(1-\rho )\beta }{\gamma _{2}}]\frac{\langle k^{2}\rangle }{\langle k\rangle }$ is the critical threshold, which means the average number of secondary infections caused by one infected individual in a susceptible population.

#### Theorem 2.1

For the system (9), the following situations hold

1. when $R_{0}<1$, the disease-free equilibrium $E^{0}(1,0,0)$ is locally asymptotically stable;
2. when $R_{0}>1$, the system has a unique epidemic equilibrium $E^{*}(s_{k}^{*},h_{k}^{*},i_{k}^{*})$ in which,

$$\begin{aligned} s_{k}^{*}=\frac{\gamma _{1}\gamma _{2}}{\beta k\theta _{h_{k}^{*}}(\rho \gamma _{2}+(1-\rho )\gamma _{1})}h_{k}^{*}, i_{k}^{*}=\frac{(1-\rho )\gamma _{1}}{\rho \gamma _{2}}h_{k}^{*}, \end{aligned}$$

$h_{k}^{*}$ has no concrete expression, but it satisfies the conditions below:

11 $$\begin{aligned} 1=h_{k}^{*}[1+\frac{\gamma _{1}}{\rho \alpha }+\frac{(1-\rho )\gamma _{1}}{\rho \gamma _{2}}+\frac{\gamma _{1}\gamma _{2}}{\beta k\theta _{h_{k}^{*}}(\rho \gamma _{2}+(1-\rho )\gamma _{1})}] \end{aligned}$$

and the disease is permanent.

The proof of the theorem is given in the supplementary, and in which

$$\begin{aligned} \theta _{h}^{*}= \sum \limits _{m=1}^{n}\frac{mP(m)h_{m}^{*}}{\langle k\rangle }, \theta _{i}^{*}= \sum \limits _{m=1}^{n}\frac{mP(m)i_{m}^{*}}{\langle k\rangle }, \theta ^{*}= \theta _{h}^{*}+\theta _{i}^{*}. \end{aligned}$$

### The periodicity in different groups

**Figure 3:**
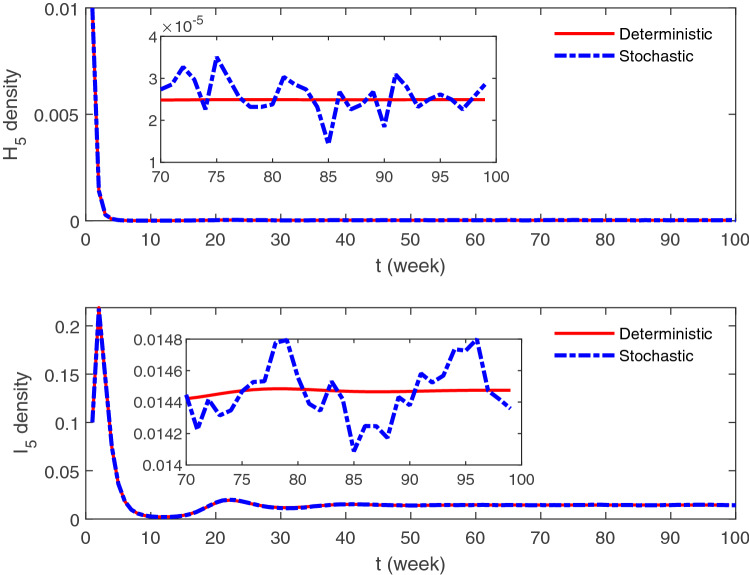
A realization of a stochastic *SHIRS* model with the degree of 5 and its deterministic counterpart. The values of parameters used in this simulation are listed in Table 2. The stochastic simulation is implemented by the event algorithm of Gillespie with transition rates listed in Table 1. The deterministic curve is generated according to the Eq. (9).

Through the above analysis, we know that for the deterministic system (9), when $R_{0}>1$, the disease ultimately fixed in the point of $E^{*}$, yet, the influenza exhibits oscillating phenomenon in reality by the influence of noise even neglecting the seasonal factor, which can be seen from the Fig. 3. Hence we use the van Kampen’s system-size expansion method to obtain the higher-order terms which can be used to investigate the perturbations around the steady-state solution of the deterministic system.

Firstly, the new continuous random variables $x_{k}, y_{k}, z_{k}$ are brought in , which have the following relationship between the discrete variables $S_{k},H_{k},I_{k}$ and the corresponding density variables $s_{k},h_{k},i_{k}$

12 $$\begin{aligned} S_{k}/N_{k}=s_{k}+x_{k}/\sqrt{N_{k}},\\ H_{k}/N_{k}=h_{k}+y_{k}/\sqrt{N_{k}},\\ I_{k}/N_{k}=i_{k}+z_{k}/\sqrt{N_{k}}. \end{aligned}$$

The variables $x_{k}, y_{k}, z_{k}$ are corrections of $s_{k},h_{k},i_{k}$, which are in ${N_{k}}^{-\frac{1}{2}}$ terms and can be viewed as the fluctuations around the epidemic equilibrium to be the order of ${N_{k}}^{-1}$, from the aspect of central-limit theorem.

Second, in order to study the property of the fluctuation, we define a new probability distribution function $\pi (x_{k},y_{k},z_{k},t)=p(S_{k},H_{k},I_{k},t)$, the following equation can be obtained by using the chain rule:

13 $$\begin{aligned} \frac{d p}{d t}=\frac{\partial \pi }{\partial t}-\sqrt{N_{k}}\frac{\partial s_{k}}{\partial t}\times \frac{\partial \pi }{\partial x_{k}}-\sqrt{N_{k}}\frac{\partial h_{k}}{\partial t}\times \frac{\partial \pi }{\partial y_{k}}-\sqrt{N_{k}}\frac{\partial i_{k}}{\partial t}\times \frac{\partial \pi }{\partial z_{k}}. \end{aligned}$$

The detailed derivation process of (13) is given in the supplementary.

By introducing the step operators below,

14 $$\begin{aligned} \varepsilon _{S_{k}}^{\pm 1}f(S_{k},H_{k},I_{k})= f(S_{k}\pm 1,H_{k},I_{k}),\\ \varepsilon _{H_{k}}^{\pm 1}f(S_{k},H_{k},I_{k})= f(S_{k},H_{k}\pm 1,I_{k}),\\ \varepsilon _{I_{k}}^{\pm 1}f(S_{k},H_{k},I_{k})= f(S_{k},H_{k},I_{k}\pm 1), \end{aligned}$$

the Eq. (6) with transition rates can be rewritten as

15 $$\begin{aligned} \frac{d p(S_{k},H_{k},I_{k},t)}{dt}&=[(\varepsilon _{S_{k}}^{+1}\varepsilon _{H_{k}}^{-1}-1) T(S_{k}-1,H_{k}+1,I_{k}|S_{k},H_{k},I_{k})\\&\quad +(\varepsilon _{S_{k}}^{+1}\varepsilon _{I_{k}}^{-1}-1)T(S_{k}-1,H_{k},I_{k}+1|S_{k},H_{k},I_{k})\\&\quad +(\varepsilon _{H_{k}}^{+1}-1)T(S_{k},H_{k}-1,I_{k}|S_{k},H_{k},I_{k})\\&\quad +(\varepsilon _{I_{k}}^{+1}-1)T(S_{k},H_{k},I_{k}-1|S_{k},H_{k},I_{k})\\&\quad +(\varepsilon _{S_{k}}^{-1}-1)T(S_{k}+1,H_{k},I_{k}|S_{k},H_{k},I_{k})] p(S_{k},H_{k},I_{k},t). \end{aligned}$$

Expanding the step operators $\varepsilon _{S_{k}}^{\pm 1}$, $\varepsilon _{H_{k}}^{\pm 1}$ and $\varepsilon _{I_{k}}^{\pm 1}$ in a power series in ${N_{k}}^{-\frac{1}{2}}$

16 $$\begin{aligned} \varepsilon _{S_{k}}^{\pm 1}=&1\pm \frac{1}{\sqrt{N_{k}}}\frac{\partial }{\partial x_{k}}+\frac{1}{2N_{k}}\frac{\partial ^{2}}{\partial x_{k}^{2}},\\ \varepsilon _{H_{k}}^{\pm 1}=&1\pm \frac{1}{\sqrt{N_{k}}}\frac{\partial }{\partial y_{k}}+\frac{1}{2N_{k}}\frac{\partial ^{2}}{\partial y_{k}^{2}},\\ \varepsilon _{I_{k}}^{\pm 1}=&1\pm \frac{1}{\sqrt{N_{k}}}\frac{\partial }{\partial z_{k}}+\frac{1}{2N_{k}}\frac{\partial ^{2}}{\partial z_{k}^{2}}, \end{aligned}$$

and then substituting the (2)–(4), (12) and (16) into (15), making a comparison with (13) order by order yields the so-called macroscopic Eq. (9) to leading order and the following Fokker-Plance equation (FPE):

17 $$\begin{aligned} \frac{\partial \pi }{\partial t}=&-A_{11}\frac{\partial (x_{k}\pi )}{\partial x_{k}}-A_{12}\frac{\partial (y_{k}\pi )}{\partial x_{k}}-A_{13}\frac{\partial (z_{k}\pi )}{\partial x_{k}}-A_{21}\frac{\partial (x_{k}\pi )}{\partial y_{k}}-A_{22}\frac{\partial (y_{k}\pi )}{\partial y_{k}}\\&-A_{23}\frac{\partial (z_{k}\pi )}{\partial y_{k}}-A_{31}\frac{\partial (x_{k}\pi )}{\partial z_{k}}-A_{32}\frac{\partial (y_{k}\pi )}{\partial z_{k}}-A_{33}\frac{\partial (z_{k}\pi )}{\partial z_{k}}+\frac{1}{2}B_{11}\frac{\partial ^{2}\pi }{\partial x_{k}^{2}}\\&+\frac{1}{2}B_{12}\frac{\partial ^{2}\pi }{\partial x_{k}y_{k}}+\frac{1}{2}B_{13}\frac{\partial ^{2}\pi }{\partial x_{k}z_{k}}+\frac{1}{2}B_{21}\frac{\partial ^{2}\pi }{\partial y_{k}x_{k}}+\frac{1}{2}B_{22}\frac{\partial ^{2}\pi }{\partial y_{k}^{2}}+\frac{1}{2}B_{23}\frac{\partial ^{2}\pi }{\partial y_{k}z_{k}}\\&+\frac{1}{2}B_{31}\frac{\partial ^{2}\pi }{\partial z_{k}x_{k}}+\frac{1}{2}B_{32}\frac{\partial ^{2}\pi }{\partial z_{k}y_{k}}+\frac{1}{2}B_{33}\frac{\partial ^{2}\pi }{\partial z_{k}^{2}}, \end{aligned}$$

in which, the matrix $A=(A_{uv})_{3\times 3}$ and $B=(B_{uv})_{3\times 3}$ are evaluated at the epidemic equilibrium $E^{*}$, whose concrete values are:

$$\begin{aligned} A=(A_{uv})_{3\times 3}= \left( \begin{array}{ccc} \frac{\partial f_{1}}{\partial s_{k}} &{} \frac{\partial f_{1}}{\partial h_{k}} &{} \frac{\partial f_{1}}{\partial i_{k}}\\ \frac{\partial f_{2}}{\partial s_{k}} &{} \frac{\partial f_{2}}{\partial h_{k}} &{} \frac{\partial f_{2}}{\partial i_{k}}\\ \frac{\partial f_{3}}{\partial s_{k}} &{} \frac{\partial f_{3}}{\partial h_{k}}&{} \frac{\partial f_{3}}{\partial i_{k}}\\ \end{array} \right) _{(E^{*})}, \end{aligned}$$

in which

$$\begin{aligned} \begin{array}{ccc} A_{11}=-\beta k\theta ^{*}-\alpha ,&{}A_{12}=-\beta s_{k}^{*}\frac{k^{2}P(k)}{\langle k\rangle }-\alpha ,&{}A_{13}=-\beta s_{k}^{*}\frac{k^{2}P(k)}{\langle k\rangle }-\alpha ,\\ A_{21}=\rho \beta k\theta ^{*},&{}A_{22}=\rho \beta s_{k}^{*}\frac{k^{2}P(k)}{\langle k\rangle }-\gamma _{1}, &{}A_{23}=\rho \beta s_{k}^{*}\frac{k^{2}P(k)}{\langle k\rangle },\\ A_{31}=(1-\rho )\beta k\theta ^{*},&{}A_{32}=(1-\rho )\beta s_{k}^{*}\frac{k^{2}P(k)}{\langle k\rangle }, &{}A_{33}=(1-\rho )\beta s_{k}^{*}\frac{k^{2}P(k)}{\langle k\rangle }-\gamma _{2}.\\ \end{array} \end{aligned}$$

and

$$\begin{aligned} B=(B_{uv})_{3\times 3}= \left( \begin{array}{ccc} B_{11} &{} B_{12} &{} B_{13} \\ B_{21} &{} B_{22} &{} B_{23} \\ B_{31} &{} B_{32} &{} B_{33} \\ \end{array} \right) _{(E^{*})}, \end{aligned}$$

in which *B* is a symmetric matrix,

$$\begin{aligned} \begin{array}{ccc} B_{11}=\beta k s_{k}^{*}\theta ^{*}+\alpha (1-s_{k}^{*}-h_{k}^{*}-i_{k}^{*}), &{}B_{12}=-\rho \beta ks_{k}^{*}\theta ^{*},\\ B_{13}=-(1-\rho )\beta ks_{k}^{*}\theta ^{*}, &{}B_{22}=\rho \beta ks_{k}^{*}\theta ^{*}+\gamma _{1}h_{k}^{*},\\ B_{23}=0, &{}B_{33}=(1-\rho )\beta ks_{k}^{*}\theta ^{*}+\gamma _{2}i_{k}^{*}. \end{array} \end{aligned}$$

which is the microscopic equation to the next-to-leading order. It is obvious that using the system-size expansion methods one can obtain the deterministic equation, which is just the leading order. For the microscopic equation, we want to describe the fluctuations about the stochastic model by using Fourier analyze. For this purpose, the following equivalent Langevin equations (LE) is fairly intuitive than the FPE (17),

18 $$\begin{aligned} \frac{dx_{k}}{dt}=A_{11}x_{k}+A_{12}y_{k}+A_{13}z_{k}+\eta _{1},\\ \frac{dy_{k}}{dt}=A_{21}x_{k}+A_{22}y_{k}+A_{23}z_{k}+\eta _{2},\\ \frac{dz_{k}}{dt}=A_{31}x_{k}+A_{32}y_{k}+A_{33}z_{k}+\eta _{3}, \end{aligned}$$

in which the stochastic variables $x_{k}, y_{k}, z_{k}$ are corrections of $s_{k}, h_{k}, i_{k},$ and $\eta _{1}, \eta _{2}, \eta _{3}$ are Gaussian white noises whose mean are zero, correlation function are

$$\begin{aligned} \langle \eta _{u}(t)\eta _{v}(t^{'})\rangle =B_{uv}\delta (t-t^{'})(u,v=1,2,3), \end{aligned}$$

in which $\delta (t-t^{'})$ denotes the Dirac delta function. The equivalence prove of LE and FPE can be found in^28^. Taking the Fourier transform of (18) gets the following results:

19 $$\begin{aligned} -iw{\tilde{x}}_{k}=A_{11}{\tilde{x}}_{k}+A_{12}{\tilde{y}}_{k}+A_{13}{\tilde{z}}_{k}+{\tilde{\eta }}_{1},\\ -iw{\tilde{y}}_{k}=A_{21}{\tilde{x}}_{k}+A_{22}{\tilde{y}}_{k}+A_{23}{\tilde{z}}_{k}+{\tilde{\eta }}_{2},\\ -iw{\tilde{z}}_{k}=A_{31}{\tilde{x}}_{k}+A_{32}{\tilde{y}}_{k}+A_{33}{\tilde{z}}_{k}+{\tilde{\eta }}_{3},\\ <{\tilde{\eta }}_{u}(w){\tilde{\eta }}_{v}(w')>=B_{uv}\delta (w-w')(u,v=1,2,3). \end{aligned}$$

in which, ${\tilde{x}}_{k}=\int ^{+\infty }_{-\infty }x_{k}(t)e^{iwt}dt$ and *i* is imaginary unit, similar to the ${\tilde{y}}_{k}$ and ${\tilde{z}}_{k}$. Because (18) is a OU process, the corresponding limits of the mean depend on the eigenvalues of *A*, when $R_{0}>1$, by means of the Fig. 3, we can see the endemic equilibrium $E^{*}$ is stable, i.e. the eigenvalues of *A* are negative, so when $t\rightarrow \infty$, $\langle {x}_{k}, {y}_{k}, {z}_{k}\rangle \rightarrow \mathbf{0}$.

Solving the equation of (19), we can obtain:

20 $$\begin{aligned} {\tilde{x}}_{k}=\frac{-(iw)^{2}{\tilde{\eta }}_{1}+(iw)C_{1}+D_{11}{\tilde{\eta }}_{1}+D_{21}{\tilde{\eta }}_{2}+D_{31}{\tilde{\eta }}_{3}}{D(w)},\\ {\tilde{y}}_{k}=\frac{-(iw)^{2}{\tilde{\eta }}_{2}+(iw)C_{2}+D_{12}{\tilde{\eta }}_{1}+D_{22}{\tilde{\eta }}_{2}+D_{32}{\tilde{\eta }}_{3}}{D(w)},\\ {\tilde{z}}_{k}=\frac{-(iw)^{2}{\tilde{\eta }}_{3}+(iw)C_{3}+D_{13}{\tilde{\eta }}_{1}+D_{23}{\tilde{\eta }}_{2}+D_{33}{\tilde{\eta }}_{3}}{D(w)},\\ \end{aligned}$$

where

$$\begin{aligned} C_{1}&=-(A_{22}+A_{33}){\tilde{\eta }}_{1}+A_{12}{\tilde{\eta }}_{2}+A_{13}{\tilde{\eta }}_{3},\\ C_{2}&=A_{21}{\tilde{\eta }}_{1}-(A_{11}+A_{33}){\tilde{\eta }}_{2}+A_{23}{\tilde{\eta }}_{3},\\ C_{3}&=A_{31}{\tilde{\eta }}_{1}+A_{32}{\tilde{\eta }}_{2}-(A_{11}+A_{22}){\tilde{\eta }}_{3}, \end{aligned}$$

$D_{uv}$ is the inverse of algebraic complement of the corresponding element $A_{uv}$ of the matrix *A*, and the expression of denominator is given by

$$\begin{aligned} D(w)&=(iw)^{3}+\mathrm {tr}{} \textit{A}(iw)^{2}+\Omega (iw)+\det \textit{A},\\ \Omega&=A_{11}A_{22}+A_{11}A_{33}+A_{22}A_{33}-A_{13}A_{31}-A_{12}A_{21}-A_{23}A_{32}, \end{aligned}$$

where tr$\textit{A}$ denotes the trace of *A* and $\det \textit{A}$ means the value of the determinant of *A*.

Averaging the squared moduli of ${\tilde{x}}_{k}$, ${\tilde{y}}_{k}$ and ${\tilde{z}}_{k}$ gives the power-spectrum:

21 $$\begin{aligned} P_{S_{k}}(w)=\langle |{\tilde{x}}_{k}|^{2}\rangle =\frac{B_{11}w^{4}+\Lambda _{1}w^{2}+\Gamma _{1}}{|D(w)|^{2}},\\ P_{H_{k}}(w)=\langle |{\tilde{y}}_{k}|^{2}\rangle =\frac{B_{22}w^{4}+\Lambda _{2}w^{2}+\Gamma _{2}}{|D(w)|^{2}},\\ P_{I_{k}}(w)=\langle |{\tilde{z}}_{k}|^{2}\rangle =\frac{B_{33}w^{4}+\Lambda _{3}w^{2}+\Gamma _{3}}{|D(w)|^{2}},\\ \end{aligned}$$

$$\begin{aligned} \Lambda _{1}\,=\,&B_{11}(A_{22}^{2}+A_{33}^{2})+B_{22}A_{12}^{2}+B_{33}A_{13}^{2}+2[B_{11}A_{22}A_{33}-B_{12}A_{12}(A_{22}+A_{33})\\&\quad -B_{13}A_{13}(A_{22}+A_{33})+B_{11}D_{11}+B_{12}D_{21}+B_{13}D_{31}],\\ \Lambda _{2}\,=\,&B_{11}A_{21}^{2}+B_{22}(A_{11}^{2}+A_{33}^{2})+B_{33}A_{23}^{2}+2[B_{13}A_{21}A_{23}\\&\quad +B_{22}A_{11}A_{33}-B_{12}A_{21}(A_{11}+A_{33})+B_{21}D_{12}+B_{22}D_{22}],\\ \Lambda _{3}\,=\,&B_{11}A_{31}^{2}+B_{22}A_{32}^{2}+B_{33}(A_{11}^{2}+A_{22}^{2})+2[B_{33}A_{11}A_{22}\\&\quad +B_{12}A_{31}A_{32}-B_{13}A_{31}(A_{11}+A_{22})+B_{31}D_{13}+B_{33}D_{33}],\\ \Gamma _{1}\,=\,&B_{11}D_{11}^{2}+B_{22}D_{21}^{2}+B_{33}D_{31}^{2}+2B_{12}D_{11}D_{21}+2B_{13}D_{11}D_{31},\\ \Gamma _{2}\,=\,&B_{11}D_{12}^{2}+B_{22}D_{22}^{2}+B_{33}D_{32}^{2}+2B_{12}D_{12}D_{22}+2B_{13}D_{12}D_{32},\\ \Gamma _{3}\,=\,&B_{11}D_{13}^{2}+B_{22}D_{23}^{2}+B_{33}D_{33}^{2}+2B_{12}D_{13}D_{23}+2B_{13}D_{13}D_{33},\\ |D(w)|^{2}&\,=\,(w^{3}-\Omega w)^{2}+(\det \textit{A}-w^{2}\mathrm{tr}{} \textit{A})^{2}. \end{aligned}$$

Up to this point, the analytical expression of PSD have been derived, because of the difficulty of the calculation in high-dimension coupled system, the concrete expressions of the epidemic equilibrium can’t be obtained, so we can analysis the frequency of different groups just depending on the numerical simulation.

## Results

In this section, we will use the influenza data of Taiyuan sentinel hospitals in the first quarter of 2013 to fit about our model in order to avoid the influence of environment changing, and then discuss the influence of heterogeneity to the periodicity of influenza.

### Estimation of parameters

Based on the statistical bulletion on national economic and social development of Taiyuan, the number of population in 2013 is 4.3 million. Removal rate can be set to $\gamma _{1}\,=\,7/3$, $\gamma _{2}\,=\,1$ and the rate of lose immunity is supposed to be $\alpha =1/52$ according to the mean disease course of influenza. The infection probability $\beta$ is an estimated parameter. As for the degree distribution, the power-law distribution usually conforms to the features of real world in most cases, hence, $P(k)=2m^2k^{-v}(m=3, v=3.5)$^29^, is used in this paper.

As far as we know, Chinese National Influenza Center (CNIC) has set up 554 sentinel hospitals all over the country. The city of Taiyuan has two of which: Taiyuan central hospital and the first affiliated hospital of Shanxi medical university. We have the influenza data from the two sentinel hospitals, yet, a small enough number compared with those in the whole city of Taiyuan, so $\rho$ as the proportion of Taiyuan influenza patients in sentinel hospitals, is a parameter to be evaluated.

**Figure 4:**
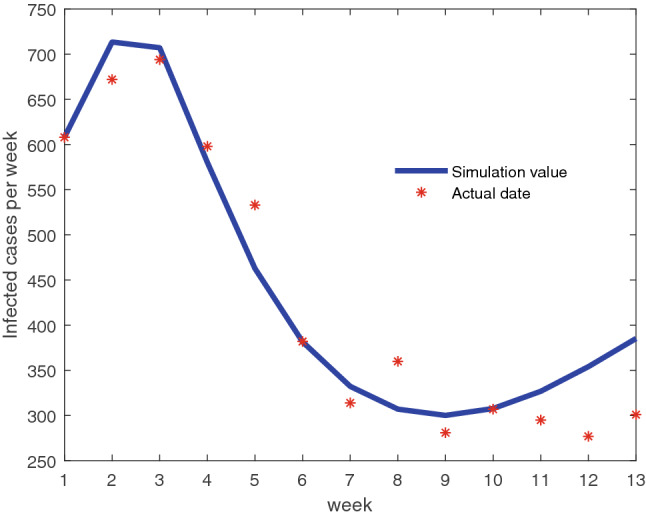
Sentinel hospitals data in the first quarter of 2013 and fitting data by using least-square are showed. The minimum (maximum) degree is 5 (500) and the mean degree is 7.5.

We adopt the least-square estimation to obtain the parameters sets $\Lambda =\{\beta ,\rho \}$, which minimizes the objective function: $Y(\Lambda )=[Y(t)-\rho N\sum \limits _{k=\min }^{\max }P(k)\beta k s_{k}\theta ]^{2}$, in which *Y*(*t*) denotes the actually influenza data from sentinel hospitals at week *t*. The Fig. 4 shows that the model data agrees well with the actual data, and the corresponding value of parameters are summarized in Table 2.

**Table 2:** Notation for model formulation and parameters value.

Parameters	Meaning	Value
*N*	The total number of people	$4.3\times 10^{6}$
$\gamma _{1}$	The recovery rate of *H*	7/3
$\gamma _{2}$	The recovery rate of *I*	1
$\alpha$	The rate of loss immunity	1/52
$\beta$	The infection probability per contact	0.8
$\rho$	The proportion of *H* in all infected people	0.004

### Prevalence of influenza in Taiyuan

CNIC has a history of more than 60 years since its foundation in 1957, especially the recently decade, the wholesome national influenza surveillance network system has made the influenza data more transparent. Our influenza data from Taiyuan sentinel hospitals are just a very small part of this system. We use the week data from 2013 to 2016 to detect whether there exists a periodic phenomenon. The data reveals a clear periodicity in the outbreaks of influenza, which can be confirmed through the wavelet power spectrum of Fig. 1. The wavelet analysis can reveal the periodic changing of a time-series, which performs the spectral characteristics as a function of time. The oscillations of influenza have a obvious annual variation, yet, the wavelet analysis based on the data shows another significant periodicity: semi-annual.

### Influence of stochasticity and network structure on influenza outbreaks

The difference between the deterministic and stochastic simulation is revealed in Fig. 3, which is a particular case of fluctuation who has been mentioned in introduction. It indicates that the demographic noise and network struction can induce rich periodic phenomenon. We can understand the influence of network struction on the expected fluctuations of influenza via our analysis.

**Figure 5:**
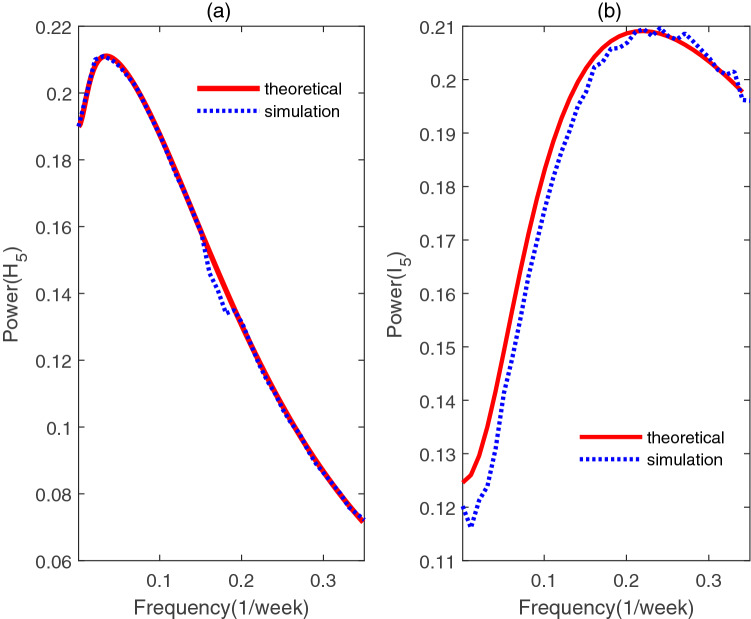
Comparisons between the theoretical prediction of PSD (21) and the average PSD obtained from the stochastic simulation, for the fluctuations of the infected with degree 5.

**Figure 6:**
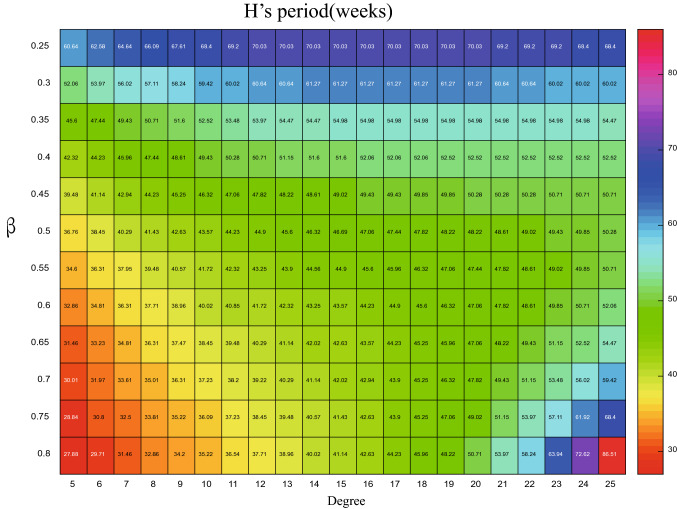
The period of *H* with different degree and $\beta$, when $\rho =0.004$.

**Figure 7:**
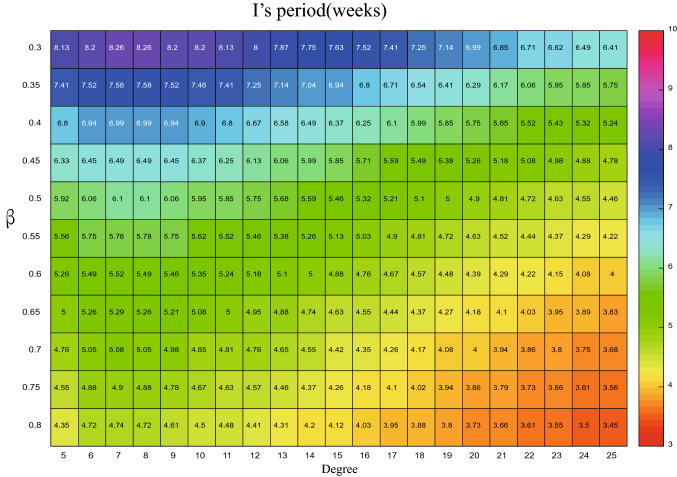
The period of *I* with different degree and $\beta$, when $\rho =0.004$.

**Figure 8:**
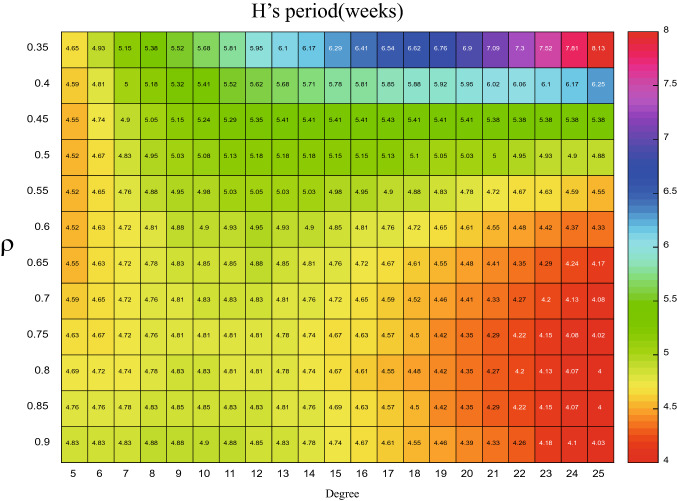
The period of *H* with different degree and $\rho$, when $\beta =0.8$.

**Figure 9:**
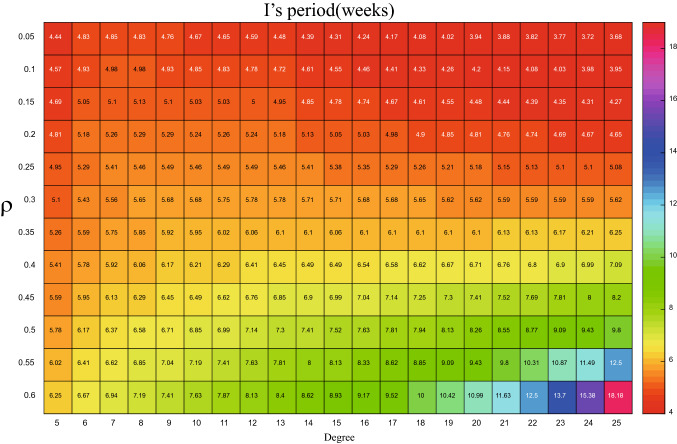
The period of *I* with different degree and $\rho$, when $\beta =0.8$.

The accuracy of the theoretical analysis consequence of PSD via (21) has been verified with the simulated results in Fig. 5, using the parameters listed in Table 2. We can go further to detect how the period of influenza varies with the changes of degree *k* by using the formula (21). Based on this, the x-coordinate (i.e. $\omega$) for the maximum value of the PSD can be obtained from the analytical expression, then the corresponding period (i.e. $1/\omega$) for different parameters are plotted in Figs. 6, 7, 8, and 9. In order to have a better visual effect, we just present the degree from 5 to 25 in these figures, on the whole, it does not affect our conclusions, because this group accounts for 99 percent of the total population.

When the $\rho =0.004$ is fixed, how the period is influenced by the degree and $\beta$ is showed in Figs. 6 and 7. In Fig. 6, for the *H* whose degree is less than 16, the period goes up as the $\beta$ decrease, yet, when the degree is large than 16, the relationship between $\beta$ and period is nonmonotonic, as $\beta$ increases, the period decreases and then increases. On the other hand, when $\beta \geqslant 0.4$, as the degree increases, so does the *H*’s period, while $\beta <0.4$, the period increases at the beginning and then decreases with the augment of the degree. There exists obvious nonlinear relationship between the *I*’s period, degree and $\beta$. For the fixed degree, increasing the $\beta$, the value of period becomes small, yet, when the $\beta$ is fixed, the relationship between the degree and *I*’s period is non-monotonous. When the degree is small than $\langle k\rangle$, the period becomes larger as the degree increases, yet, the trend is opposite on the other side of $\langle k\rangle$, which can be obtained from the Fig. 7. This means that, for the infected people *I* whose degree more close to $\langle k\rangle$, the more safer.

When $\beta =0.8$ is fixed, the relationship between the period, $\rho$ and degree is showed in Figs. 8 and 9. From Fig. 8 we can observe that when $\rho \leqslant 0.4$, for the infected people of *H*, the degree is more bigger, the period is more larger, which means that people are more safer, yet, when $\rho >0.4$, with the degree increasing, the period increases firstly and decreases later, which means the people of *H* whose degree located intermediate is more safer. This is quite opposite for *I* in Fig. 9. On the other hand, when *H*’s degree is fixed, the period decreases at the beginning and then increases as the $\rho$ increasing. This phenomenon from a side explains that the importance of the medical resources. For the infected people of *I*, the relationship is different in Fig. 9. With the increase of $\rho$, which means more and more infected people go to the sentinel hospitals, leading the *I* becomes more and more safer, i.e. the period is more and more longer, this change is even more pronounced for *I* with higher degree.

## Discussion

In this paper, we have investigated the effect of network structure on the frequency of the influenza outbreaks, focusing on the power-law networks, by adapting the van Kampen expansion approach from *SHIRS* disease to develop a fully stochastic model. This leads to a reasonable explanation for the periodic phenomena of influenza, and helps us to understand the complex fluctuations of influenza in Taiyuan with a dominant period of half years without the external factors of seasonal.

We can have a better understanding of the interaction between the deterministic and stochastic components of the system by using the method of van Kampen expansion. The analytical solution of PSD agrees well with the simulation data. The result reveals that for the infected people who go to the sentinel hospitals, i.e. *H*, the relationship between the $\rho$, $\beta$ and period is nonlinear, even for the hospitals hospitalization rate $\rho$, just an appropriate one is benefit for the patients. On the other hand, the nonlinear relationship between the three is also suitable for the infected people *I* in most cases, except that the relationship between $\rho$ (or $\beta$) and period is monotonous when other parameters are fixed.

Our work emphasizes the importance of heterogeneous contact network on the periodic outbreak of influenza, which further validates the truth that the combined action of the non-linear system and stochasticity may results many novel phenomena. On the other hand, our study also show that there exists a very close relation between the phenomenon of multi-cycle and the heterogeneity of contact.

### Limitations

To obtain the specific analytical solutions are difficult for the high dimensional coupling system, so we can not give the explicit formula about the relationship between the period, degree, $\beta$ and $\rho$, and just have a intuitive understanding from the numerical simulation.

The influenza date we obtained from the sentinel hospitals are weekly, so we set the time-boxed intervals measured in week. Comparing to the daily one, the weekly contact network will be not very precise and may cause the repeatedly contact problem, which one we omitted in this paper.

The approach of van Kampen is limited to the following two conditions: a formal treatment of demographic stochasticity; the existence of stable non-zero solution, for simpleness we do not take into account the changing population, seasonal forcing and the correlation between the degree. In essence, the migration of population and behavioral changing in flu season is unavoidable, which is missing in our work.

### Possible extensions

It is easy to adapt this method to many diseases exhibited fluctuations, such as malaria, cholera, hand foot and mouth disease and so on. Especially for childhood diseases, similar study may gives a good idea on avoiding frequent illness. At the same time, if there are several strains that coexist for a disease, such as influenza, our method may also deduce the period for each strain, which may be good news for the vaccine’s manufacturers.

**Figure 10:**
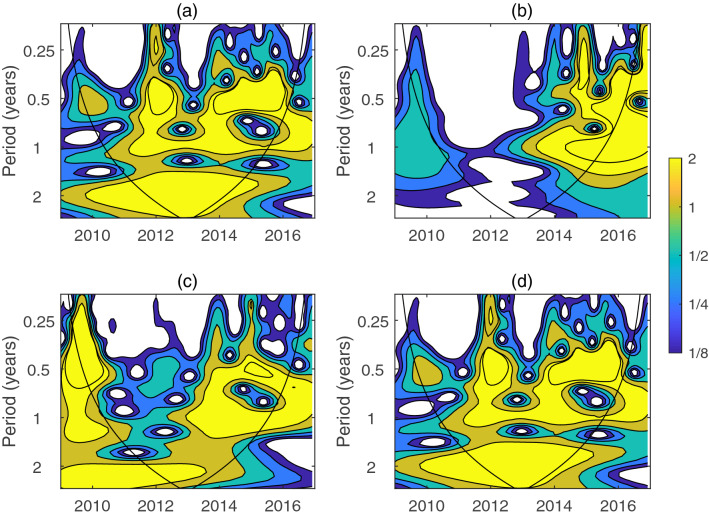
The results of wavelet analysis, where the monthly influenza data of (**a**) Shanghai, (**b**) Beijing, (**c**) Jiangsu province and (**d**) Tianjin obtained from CDC since 2009–2016.

We can find that from Fig. 10, even among the very nearest cities, like Beijing and Tianjin, Jiangsu and Shanghai, though the climate differences can be ignored, the wavelet power spectrum is greatly different. This may prove the importance of the contact network. On the other hand, even though the climate characteristics of city are different from north part of China (Beijing and Tianjin) to the south part (Jiangsu and Shanghai), it is a surprise phenomenon that Tianjin and Shanghai exhibit the similar periodic law. Furthermore, the same method can also be used to the influenza data of tropical cities, then comparisons with existing temperate data may reveal the dominant factors. Which one is the key factor lead to this? Temperature, humidity, wind speed, haze or human behavior? It deserves our further investigation.

## Supplementary Information

Supplementary Information 1.

## References

[1] Colizza V, Barrat A, Barthelemy M, Vespignani A (2006). The role of the airline transportation network in the prediction and predictability of global epidemics. Proc. Natl. Acad. Sci..

[2] Pei S, Kandula S, Yang W, Shaman J (2017). Forecasting the spatial transmission of influenza in the united states. Proc. Natl. Acad. Ences USA.

[3] Merler S, Ajelli M (2010). The role of population heterogeneity and human mobility in the spread of pandemic influenza. Proc. Royal Soc. B Biol. Sci..

[4] Colijn C (2010). What is the mechanism for persistent coexistence of drug-susceptible and drug-resistant strains of streptococcus pneumoniae. J. Royal Soc. Interface.

[5] Dietz K, Heesterbeek JAP (2002). Daniel bernoullis epidemiological model revisited. Math. Biosci..

[6] EnKo PD (1989). On the course of epidemics of some infectious diseases. Int. J. Epidemiol..

[7] Bacar N (2011). A Short History of Mathematical Population Dynamics.

[8] Kermack AG, McKendrick WO (1927). A contribution to the mathematical theory of epidemics. Proc. Royal Soc. Math. Phys. Eng. Ences.

[9] Newman MEJ (2010). Networks: An Introduction.

[10] Keeling MJ, Eames KTD (2005). Networks and epidemic models. J. Royal Soc. Interface.

[11] Pastor-Satorras R, Vespignani A (2001). Epidemic spreading in scale-free networks. Phys. Rev. Lett..

[12] Peng X, Xu XJ, Small M, Fu X, Jin Z (2016). Prevention of infectious diseases by public vaccination and individual protection. J. Math. Biol..

[13] Miller JC (2011). A note on a paper by erik volz: Sir dynamics in random networks. J. Math. Biol..

[14] Pastor-Satorras R, Vespignani A (2001). Epidemic dynamics and endemic states in complex networks. Phys. Rev. E.

[15] Lindquist J, Ma J, Driessche PVD, Willeboordse FH (2011). Effective degree network disease models. J. Math. Biol..

[16] Stone L, Olinky R, Huppert A (2007). Seasonal dynamics of recurrent epidemics. Nature.

[17] Rohani NP (2008). Noise, nonlinearity and seasonality: The epidemics of whooping cough revisited. J. Royal Soc. Interface.

[18] Ponciano JM, Capistrán MA, Pascual M (2011). First principles modeling of nonlinear incidence rates in seasonal epidemics. PLoS Comput. Biol..

[19] Keeling MJ, Rohani P, Grenfell BT (2001). Seasonally forced disease dynamics explored as switching between attractors. Phys. D Nonlinear Phenomena.

[20] Alonso D, Mckane AJ (2002). Extinction dynamics in mainland-island metapopulations: An n-patch stochastic model. Bull. Math. Biol..

[21] Chaffee J, Kuske R (2011). The effect of loss of immunity on noise-induced sustained oscillations in epidemics. Bull. Math. Biol..

[22] Earn D (2000). A simple model for complex dynamical transitions in epidemics. Science.

[23] Van Kampen, N. G. *Stochastic Processes in Physics and Chemistry (Third Edition)* (The Netherlands:Elsevier, 1992).

[24] Alonso D, Mckane AJ, Pascual M (2007). Stochastic amplification in epidemics. J. Royal Soc. Interface.

[25] Wang RH, Jin Z, Liu QX, Koppel JVD, Alonso D (2012). A simple stochastic model with environmental transmission explains multi-year periodicity in outbreaks of avian flu. PLoS ONE.

[26] Torrence Christopher, Compo Gilbert P (1998). A practical guide to wavelet analysis. Bull. Am. Meteorol. Soc..

[27] Cazelles B, Chavez M, De Magny GC, Guegan J, Hales S (2007). Time-dependent spectral analysis of epidemiological time-series with wavelets. J. Royal Soc. Interface.

[28] Gillespie DT (1976). A general method for numerically simulating the stochastic time evolution of coupled chemical reactions. J. Comput. Phys..

[29] Jin Z (2011). Modelling and analysis of influenza a (h1n1) on networks. BMC Public Health.

