# Supplementary material for: A stochastic model explains the periodicity phenomenon of influenza on network
Source: Sci Rep. 2021 Oct 25;11:20996. doi: 10.1038/s41598-021-00260-3 (PMC8546073; doi:10.1038/s41598-021-00260-3)
Supplement: Supplementary file 1 — Supplementary Information 1. [file 41598_2021_260_MOESM1_ESM.pdf]

A stochastic model explains the periodicity phenomenon of influenza on network

Hong Yang[1,2] **and Zhen Jin**[1,2,*]

[1]Complex System Research Center, Shanxi University, Taiyuan 030006, Shanxi, China
[2]Shanxi Key Laboratory of Mathematical Techniques and Big Data Analysis on Disease Control and Prevention, Shanxi University, Taiyuan 030006, Shanxi, China
*jinzhn@263.net

The proof of the theorem 2.1

First, we prove the locally asymptotically stability of disease-free equilibrium E^0, when $R_0 < 1$.

According to the reference[1], we can obtain the reproduction number by using the methods of next-generation matrix: $R_0 = \rho(FV^{-1})$, (i.e. the spectral radius of the matrix FV^{-1}), in which F denotes the rate of appearance of new infections and V denotes the rate of transfer of individuals out of the compartments. To calculate the reproduction number, we first give the concrete expressions of F and V,

$$F = \begin{pmatrix} F_{11}^{n\times n} & F_{12}^{n\times n} \\ F_{21}^{n\times n} & F_{22}^{n\times n} \end{pmatrix},$$

where

$$F_{11}^{n\times n} = F_{12}^{n\times n} = \frac{\rho\beta}{\langle k\rangle} \begin{pmatrix} P(1) & 2P(2) & \cdots & nP(n) \\ 2P(1) & 2^2P(2) & \cdots & 2nP(n) \\ \vdots & \vdots & \vdots & \vdots \\ nP(1) & 2nP(2) & \cdots & n^2P(n) \end{pmatrix},$$

$$F_{21}^{n\times n} = F_{22}^{n\times n} = \frac{(1-\rho)\beta}{\langle k\rangle} \begin{pmatrix} P(1) & 2P(2) & \cdots & nP(n) \\ 2P(1) & 2^2P(2) & \cdots & 2nP(n) \\ \vdots & \vdots & \vdots & \vdots \\ nP(1) & 2nP(2) & \cdots & n^2P(n) \end{pmatrix},$$

$$V = -\begin{pmatrix} -\gamma_1 & \cdots & 0 & 0 & \cdots & 0 \\ \vdots & \vdots & \vdots & \vdots & \vdots & \vdots \\ 0 & \cdots & -\gamma_1 & 0 & \cdots & 0 \\ 0 & \cdots & 0 & -\gamma_2 & \cdots & 0 \\ \vdots & \vdots & \vdots & \vdots & \vdots & \vdots \\ 0 & \cdots & 0 & 0 & \cdots & -\gamma_2 \end{pmatrix},$$

Setting $C = FV^{-1}$, we have

$$C = \begin{pmatrix} \frac{1}{\gamma_1}F_{11}^{n\times n} & \frac{1}{\gamma_2}F_{12}^{n\times n} \\ \frac{1}{\gamma_1}F_{21}^{n\times n} & \frac{1}{\gamma_2}F_{22}^{n\times n} \end{pmatrix}.$$

Then we can obtain the reproductive number

$$R_0 = \mathrm{trC} = [\frac{\rho\beta}{\gamma_1} + \frac{(1-\rho)\beta}{\gamma_2}]\frac{\langle \mathrm{k}^2\rangle}{\langle \mathrm{k}\rangle}.$$

Hence, it is obvious to know that the trivial point E^0 is locally asymptotically stable if and only if $R_0 < 1$.

Second, when $R_0 > 1$, before proving the stability of the endemic equilibrium point, we must first prove its existence. To simplify writing, define

$$b_1 = \rho\gamma_2 + (1-\rho)\gamma_1, b_2 = \rho\alpha\gamma_2 + \rho\gamma_1\gamma_2 + \rho(1-\rho)\alpha\gamma_1.$$

Condition (11) that the equilibrium point of the system (9) satisfies is equivalent to

$$h_k^* = \frac{\rho\alpha\gamma_2\beta k\theta_{h_k^*}b_1}{\beta k\theta_{h_k^*}b_1b_2 + \rho\alpha\gamma_1\gamma_2^2}.$$

Using the expression (10) of θ_h, we can get the following equation:

$$\theta_{h_k^*} = \frac{1}{\langle k\rangle}\sum_{m=1}^{n}\frac{mP(m)\rho\alpha\gamma_2\beta m\theta_{h_k^*}b_1}{\beta m\theta_{h_k^*}b_1b_2 + \rho\alpha\gamma_1\gamma_2^2},$$

In order to obtain the condition of the existence and uniqueness of positive solution, we define the following function:

$$F(\theta_h) = \theta_h - \frac{1}{\langle k\rangle}\sum_{m=1}^{n}\frac{mP(m)\rho\alpha\gamma_2\beta m\theta_h b_1}{\beta m\theta_h b_1b_2 + \rho\alpha\gamma_1\gamma_2^2},$$

in which $\theta_h \in [0,1]$. The first and second derivatives of the function can be derived

$$\frac{dF(\theta_h)}{d\theta_h} = 1 - \frac{1}{\langle k\rangle}\sum_{m=1}^{n}\frac{m^2P(m)\beta\rho^2\alpha^2\gamma_1\gamma_2^3b_1}{[\beta m\theta_h b_1b_1 + \rho\alpha\gamma_1\gamma_2^2]^2},$$

$$\frac{d^2F(\theta_h)}{d\theta_h^2} = \frac{2}{\langle k\rangle}\sum_{m=1}^{n}\frac{m^3P(m)\beta^2\rho^2\alpha^2\gamma_1\gamma_2^3b_1^2b_2}{[\beta m\theta_h b_1b_2 + \rho\alpha\gamma_1\gamma_2^2]^3}.$$

Because $F(\theta_h)$ is a concave function, and $F(0) = 0, F(1) > 0$, so, the sufficient and necessary condition for the function to have a unique positive solution in the interval [0,1] is

$$\frac{dF(\theta_h)}{d\theta_h}|_{\theta_h=0} = 1 - R_0 < 0.$$

It is obvious that when the basic reproductive number $R_0 > 1$, the system (9) has a only positive equilibrium point.

Similar to the[2], we can prove that the disease is permanent if $R_0 > 1$, i.e. there exists a $\zeta > 0$, such that $\liminf_{t\to\infty}\sum_{k=1}^{n}P(k)(h_k + i_k) > 0$ by using the Theorem 4.6 in[3]. Define

$$M = \{(s_1,h_1,i_1,...,s_n,h_n,i_n) : s_k,h_k,i_k \geq 0, s_k + h_k + i_k \leq 1, k = 1,2,...,n\},$$

$$M_0 = \{(s_1,h_1,i_1,...,s_n,h_n,i_n) \in M : \sum_{k=1}^{n}P(k)(h_k + i_k) > 0\}, \partial M_0 = M\backslash M_0.$$

Obviously, M is positively invariant of the system.

If $s_k(0) \geq 0$ and $\sum_{k=1}^{n}P(k)(h_k(0) + i_k(0)) > 0$ for $k = 1,2,...,n$, then $s_k(t) \geq 0$ and $\sum_{k=1}^{n}P(k)(h_k(t) + i_k(t)) > 0$ for all $t > 0$. Since

$$(\sum_{k=1}^{n}P(k)(h_k(t) + i_k(t)))' \geq -\gamma\sum_{k=1}^{n}P(k)(h_k(t) + i_k(t)),$$

in which $\gamma = \max\{\gamma_1,\gamma_2\}$, and $\sum_{k=1}^{n}P(k)(h_k(0) + i_k(0)) > 0$, we have

$$\sum_{k=1}^{n}P(k)(h_k(t) + i_k(t)) \geq \sum_{k=1}^{n}P(k)(h_k(0) + i_k(0))e^{\gamma t} > 0.$$

Thus, M_0 is also positively invariant of the system.

Furthermore, there exists a compact set B which satisfied the compactness condition (C4.2) in[3] making all the solutions of the system initiated in M remain this forever. Denote

$$\begin{aligned} M_\partial = &\{(s_1(0),h_1(0),i_1(0),...,s_n(0),h_n(0),i_n(0)) : \\ &(s_1(t),h_1(t),i_1(t),...,s_n(t),h_n(t),i_n(t)) \in \partial M_0, t \geq 0\}, \\ \Omega = &\cup\{\omega(y) : y \in M_\partial\}, \end{aligned}$$

where $\omega(y)$ means the omega limit set of the solutions starting from y.
Limiting the system on M_∂ gives

$$\begin{cases} \frac{ds_k}{dt} = \alpha(1-s_k-h_k-i_k), \\ \frac{dh_k}{dt} = -\gamma_1 h_k, \\ \frac{di_k}{dt} = -\gamma_2 i_k. \end{cases} \tag{S.1}$$

It is obvious that the system (S.1) has a unique equilibrium E^0 in M, and it is same for the set M_∂, since the E^0 is locally asymptotically stable, it is globally asymptotically stable for the linear system (S.1). So E^0 is a isolated and acyclic covering of Ω, i.e. $\Omega = \{E^0\}$.

Finally, if we can prove the E^0 is a weak repeller for M_0, then

$$\limsup_{t\to\infty} \mathrm{dist}(E^0, (s_1(t), h_1(t), i_1(t), ..., s_n(t), h_n(t), i_n(t))) > 0,$$

where $(s_1(t), h_1(t), i_1(t), ..., s_n(t), h_n(t), i_n(t))$ denotes an arbitrarily solution starting from the set M_0, we can end our prove. According to the Lemma 3.5 in[4], we only need to certify the stable manifold of E^0 (i.e. $W^s(E^0)$) has no intersection with M_0, i.e. $W^s(E^0) \cap M_0 = \emptyset$. Suppose it is wrong, then there exists a solution $(s_1(t), h_1(t), i_1(t), ..., s_n(t), h_n(t), i_n(t)) \in M_0$, s.t.

$$s_k(t) \to 1, h_k(t) \to 0, i_k(t) \to 0, \text{when t} \to \infty, \tag{S.2}$$

Since $R_0 = [\frac{\rho\beta}{\gamma_1} + \frac{(1-\rho)\beta}{\gamma_2}]\frac{\langle k^2\rangle}{\langle k\rangle} > 1$, there exists $\eta > 0$, s.t. $\frac{\beta}{\gamma}\frac{\langle k^2\rangle}{\langle k\rangle}(1-\eta) > 1$.
According to the (S.2), there exists $T > 0$, for all $t > T$, s.t.

$$1-\eta < s_k(t) < 1, 0 < h_k(t), i_k(t) < \eta, k = 1, 2, ..., n.$$

Denote

$$V(t) = \sum_{k=1}^{n} kP(k)(h_k(t) + i_k(t)),$$

Along the solution $(s_1(t), h_1(t), i_1(t), ..., s_n(t), h_n(t), i_n(t))$, we can get the derivative of V:

$$\begin{aligned} V(\dot{t}) &= \frac{\sum_{k=1}^{n} k^2 P(k) s_k(t) V(t)}{\langle k\rangle} - \gamma_1 \sum_{k=1}^{n} kP(k) h_k(t) - \gamma_2 \sum_{k=1}^{n} kP(k) i_k(t) \\ &\geq [\frac{\langle k^2\rangle}{\langle k\rangle}(1-\eta)\beta - \gamma_1] \sum_{k=1}^{n} kP(k) h_k(t) + [\frac{\langle k^2\rangle}{\langle k\rangle}(1-\eta)\beta - \gamma_2] \sum_{k=1}^{n} kP(k) i_k(t) \\ &\geq \rho V(t), \end{aligned}$$

where

$$\rho = \frac{\beta}{\gamma}\frac{\langle k^2\rangle}{\langle k\rangle}(1-\eta) > 0.$$

Hence $\lim_{t\to\infty} V(t) = \infty$, which contradicts to the boundness of $V(t)$.

The derived process of (13)

Because of $\pi(x_k, y_k, z_k, t) = p(S_k, H_k, I_k, t)$, at the epidemic equilibrium E^* takes the total derivative on both sides leading to:

$$\begin{aligned} \frac{dp}{dt} &= \frac{\partial \pi}{\partial t} + \frac{\partial \pi}{\partial x_k}\frac{\partial x_k}{\partial t} + \frac{\partial \pi}{\partial y_k}\frac{\partial y_k}{\partial t} + \frac{\partial \pi}{\partial z_k}\frac{\partial z_k}{\partial t} \\ &= \frac{\partial \pi}{\partial t} + \frac{\partial \pi}{\partial x_k}\frac{\partial x_k}{\partial s_k}\frac{\partial s_k}{\partial t} + \frac{\partial \pi}{\partial y_k}\frac{\partial y_k}{\partial h_k}\frac{\partial h_k}{\partial t} + \frac{\partial \pi}{\partial y_k}\frac{\partial z_k}{\partial i_k}\frac{\partial i_k}{\partial t} \\ &= \frac{\partial \pi}{\partial t} - \sqrt{N_k}\frac{\partial s_k}{\partial t} \times \frac{\partial \pi}{\partial x_k} - \sqrt{N_k}\frac{\partial h_k}{\partial t} \times \frac{\partial \pi}{\partial y_k} - \sqrt{N_k}\frac{\partial i_k}{\partial t} \times \frac{\partial \pi}{\partial z_k}. \end{aligned}$$

The deduce process of (17)

Because of

$$\begin{aligned}\varepsilon_{S_k}^{+1}\varepsilon_{A_k}^{-1}-1=&(1+\frac{1}{\sqrt{N_k}}\frac{\partial}{\partial x_k}+\frac{1}{2N_k}\frac{\partial^2}{\partial x_k^2})(1-\frac{1}{\sqrt{N_k}}\frac{\partial}{\partial y_k}+\frac{1}{2N_k}\frac{\partial^2}{\partial y_k^2})-1\\=&\frac{1}{\sqrt{N_k}}\frac{\partial}{\partial x_k}+\frac{1}{2N_k}\frac{\partial^2}{\partial x_k^2}-\frac{1}{\sqrt{N_k}}\frac{\partial}{\partial y_k}+\frac{1}{2N_k}\frac{\partial^2}{\partial y_k^2}-\frac{1}{N_k}\frac{\partial^2}{\partial x_k\partial y_k}+O(\frac{\partial^2}{\partial x_k\partial y_k})+O(\frac{\partial^2}{\partial x_k^2})+O(\frac{\partial^2}{\partial y_k^2}),\end{aligned}\tag{S.3}$$

and

$$\begin{aligned}T(S_k-1,A_k+1,I_k|S_k,A_k,I_k)\pi&=\rho\beta kS_k\frac{\sum\limits_{m=1}^{n}(mA_m+mI_m)}{\sum\limits_{m=1}^{n}mN_m}\pi\\&=\rho\beta k(N_ks_k+\sqrt{N_k}x_k)\frac{\sum\limits_{m=1}^{n}m(N_ma_m+\sqrt{N_m}y_m+N_mi_m+\sqrt{N_m}z_m)}{\sum\limits_{m=1}^{n}mN_m}\pi\\&=\rho\beta\frac{k}{\langle k\rangle}(N_ks_k+\sqrt{N_k}x_k)\pi\sum_{m=1}^{n}mP(m)(a_m+i_m+\frac{y_m+z_m}{\sqrt{N_m}}),\end{aligned}\tag{S.4}$$

then for each of the terms in (S.3) $*$ (S.4), the specific calculation procedure will be given in the following in turn.

$$\begin{aligned}&\frac{1}{\sqrt{N_k}}\frac{\partial}{\partial x_k}(\rho\beta\frac{k}{\langle k\rangle}N_ks_k\pi\sum_{m=1}^{n}mP(m)(a_m+i_m+\frac{y_m+z_m}{\sqrt{N_m}}))\\=&\sqrt{N_k}\rho\beta\frac{k}{\langle k\rangle}s_k(\sum_{m=1}^{n}mP(m)(a_m+i_m)+\sum_{m\neq k}mP(m)\frac{y_m+z_m}{\sqrt{N_m}})\frac{\partial\pi}{\partial x_k}+\rho\beta s_k\frac{k^2P(k)}{\langle k\rangle}(\frac{\partial(y_k\pi)}{\partial x_k}+\frac{\partial(z_k\pi)}{\partial x_k}),\\&\frac{1}{\sqrt{N_k}}\frac{\partial}{\partial x_k}(\rho\beta\frac{k}{\langle k\rangle}\sqrt{N_k}x_k\pi\sum_{m=1}^{n}mP(m)(a_m+i_m+\frac{y_m+z_m}{\sqrt{N_m}}))\\=&\rho\beta\frac{k}{\langle k\rangle}(\sum_{m=1}^{n}mP(m)(a_m+i_m)+\sum_{m\neq k}mP(m)\frac{y_m+z_m}{\sqrt{N_m}})\frac{\partial(x_k\pi)}{\partial x_k}+\frac{1}{\sqrt{N_k}}\rho\beta\frac{k^2P(k)}{\langle k\rangle}(\frac{\partial(x_ky_k\pi)}{\partial x_k}+\frac{\partial(x_kz_k\pi)}{\partial x_k}),\\&\frac{1}{\sqrt{N_k}}\frac{\partial}{\partial y_k}(\rho\beta\frac{k}{\langle k\rangle}N_ks_k\pi\sum_{m=1}^{n}mP(m)(a_m+i_m+\frac{y_m+z_m}{\sqrt{N_m}}))\\=&\sqrt{N_k}\rho\beta\frac{k}{\langle k\rangle}s_k(\sum_{m=1}^{n}mP(m)(a_m+i_m)+\sum_{m\neq k}mP(m)\frac{y_m+z_m}{\sqrt{N_m}})\frac{\partial\pi}{\partial y_k}+\rho\beta s_k\frac{k^2P(k)}{\langle k\rangle}(\frac{\partial(y_k\pi)}{\partial y_k}+\frac{\partial(z_k\pi)}{\partial y_k}),\\&\frac{1}{\sqrt{N_k}}\frac{\partial}{\partial y_k}(\rho\beta\frac{k}{\langle k\rangle}\sqrt{N_k}x_k\pi\sum_{m=1}^{n}mP(m)(a_m+i_m+\frac{y_m+z_m}{\sqrt{N_m}}))\\=&\rho\beta\frac{k}{\langle k\rangle}(\sum_{m=1}^{n}mP(m)(a_m+i_m)+\sum_{m\neq k}mP(m)\frac{y_m+z_m}{\sqrt{N_m}})\frac{\partial(x_k\pi)}{\partial y_k}+\frac{1}{\sqrt{N_k}}\rho\beta\frac{k^2P(k)}{\langle k\rangle}(\frac{\partial(x_ky_k\pi)}{\partial y_k}+\frac{\partial(x_kz_k\pi)}{\partial y_k}),\\&\frac{1}{2N_k}\frac{\partial^2}{\partial x_k^2}(\rho\beta\frac{k}{\langle k\rangle}N_ks_k\pi\sum_{m=1}^{n}mP(m)(a_m+i_m+\frac{y_m+z_m}{\sqrt{N_m}}))\\=&\frac{1}{2}\rho\beta\frac{k}{\langle k\rangle}s_k(\sum_{m=1}^{n}mP(m)(a_m+i_m)+\sum_{m\neq k}mP(m)\frac{y_m+z_m}{\sqrt{N_m}})\frac{\partial^2\pi}{\partial x_k^2}+\frac{1}{2\sqrt{N_k}}\rho\beta s_k\frac{k^2P(k)}{\langle k\rangle}(\frac{\partial^2(y_k\pi)}{\partial x_k^2}+\frac{\partial^2(z_k\pi)}{\partial x_k^2}),\end{aligned}$$

$$\frac{1}{2N_k}\frac{\partial^2}{\partial x_k^2}(\rho\beta\frac{k}{\langle k\rangle}\sqrt{N_k}x_k\pi\sum_{m=1}^{n}mP(m)(a_m+i_m+\frac{y_m+z_m}{\sqrt{N_m}}))$$
$$=\frac{1}{2\sqrt{N_k}}\rho\beta\frac{k}{\langle k\rangle}(\sum_{m=1}^{n}mP(m)(a_m+i_m)+\sum_{m\neq k}mP(m)\frac{y_m+z_m}{\sqrt{N_m}})\frac{\partial^2(x_k\pi)}{\partial x_k^2}+\frac{1}{2N_k}\rho\beta\frac{k^2P(k)}{\langle k\rangle}(\frac{\partial^2(x_ky_k\pi)}{\partial x_k^2}+\frac{\partial^2(x_kz_k\pi)}{\partial x_k^2}),$$
$$\frac{1}{2N_k}\frac{\partial^2}{\partial y_k^2}(\rho\beta\frac{k}{\langle k\rangle}N_ks_k\pi\sum_{m=1}^{n}mP(m)(a_m+i_m+\frac{y_m+z_m}{\sqrt{N_m}}))$$
$$=\frac{1}{2}\rho\beta\frac{k}{\langle k\rangle}s_k(\sum_{m=1}^{n}mP(m)(a_m+i_m)+\sum_{m\neq k}mP(m)\frac{y_m+z_m}{\sqrt{N_m}})\frac{\partial^2\pi}{\partial y_k^2}+\frac{1}{2N_k}\rho\beta s_k\frac{k^2P(k)}{\langle k\rangle}(\frac{\partial^2(y_k\pi)}{\partial y_k^2}+\frac{\partial^2(z_k\pi)}{\partial y_k^2}),$$
$$\frac{1}{2N_k}\frac{\partial^2}{\partial y_k^2}(\rho\beta\frac{k}{\langle k\rangle}\sqrt{N_k}x_k\pi\sum_{m=1}^{n}mP(m)(a_m+i_m+\frac{y_m+z_m}{\sqrt{N_m}}))$$
$$=\frac{1}{2\sqrt{N_k}}\rho\beta\frac{k}{\langle k\rangle}(\sum_{m=1}^{n}mP(m)(a_m+i_m)+\sum_{m\neq k}mP(m)\frac{y_m+z_m}{\sqrt{N_m}}\sqrt{N_m})\frac{\partial^2(x_k\pi)}{\partial y_k^2}+\frac{1}{2N_k}\rho\beta\frac{k^2P(k)}{\langle k\rangle}(\frac{\partial^2(x_ky_k\pi)}{\partial y_k^2}+\frac{\partial^2(x_kz_k\pi)}{\partial y_k^2}),$$
$$\frac{1}{N_k}\frac{\partial^2}{\partial x_k\partial y_k}(\rho\beta\frac{k}{\langle k\rangle}N_ks_k\pi\sum_{m=1}^{n}mP(m)(a_m+i_m+\frac{y_m+z_m}{\sqrt{N_m}}))$$
$$=\rho\beta\frac{k}{\langle k\rangle}s_k(\sum_{m=1}^{n}mP(m)(a_m+i_m)+\sum_{m\neq k}mP(m)\frac{y_m+z_m}{\sqrt{N_m}})\frac{\partial^2\pi}{\partial x_k\partial y_k}+\frac{1}{\sqrt{N_k}}\rho\beta s_k\frac{k^2P(k)}{\langle k\rangle}(\frac{\partial^2(y_k\pi)}{\partial x_k\partial y_k}+\frac{\partial^2(z_k\pi)}{\partial x_k\partial y_k}),$$
$$\frac{1}{N_k}\frac{\partial^2}{\partial x_k\partial y_k}(\rho\beta\frac{k}{\langle k\rangle}\sqrt{N_k}x_k\pi\sum_{m=1}^{n}mP(m)(a_m+i_m+\frac{y_m+z_m}{\sqrt{N_m}}))$$
$$=\frac{1}{\sqrt{N_k}}\rho\beta\frac{k}{\langle k\rangle}(\sum_{m=1}^{n}mP(m)(a_m+i_m)+\sum_{m\neq k}mP(m)\frac{y_m+z_m}{\sqrt{N_m}})\frac{\partial^2(x_k\pi)}{\partial x_k\partial y_k}+\frac{1}{N_k}\rho\beta\frac{k^2P(k)}{\langle k\rangle}(\frac{\partial^2(x_ky_k\pi)}{\partial x_k\partial y_k}+\frac{\partial^2(x_kz_k\pi)}{\partial x_k\partial y_k}),$$

Since $\lim_{N_k\to\infty}\frac{1}{N_k}=0$, $\lim_{\sqrt{N_k}\to\infty}\frac{1}{\sqrt{N_k}}=0$, many terms can be ignored, then the first term on the right-hand side of (15) equal to:

$$(\varepsilon_{S_k}^{+1}\varepsilon_{A_k}^{-1}-1)T(S_k-1,A_k+1,I_k|S_k,A_k,I_k)p(S_k,A_k,I_k,t)$$
$$=\sqrt{N_k}\rho\beta\frac{k}{\langle k\rangle}s_k\sum_{m=1}^{n}mP(m)(a_m+i_m)\frac{\partial\pi}{\partial x_k}+\rho\beta\frac{k}{\langle k\rangle}\sum_{m=1}^{n}mP(m)(a_m+i_m)\frac{\partial(x_k\pi)}{\partial x_k}-\sqrt{N_k}\rho\beta\frac{k}{\langle k\rangle}s_k\sum_{m=1}^{n}mP(m)(a_m+i_m)\frac{\partial\pi}{\partial y_k}$$
$$-\rho\beta s_k\frac{k^2P(k)}{\langle k\rangle}(\frac{\partial(y_k\pi)}{\partial y_k}+\frac{\partial(z_k\pi)}{\partial y_k})-\rho\beta\frac{k}{\langle k\rangle}\sum_{m=1}^{n}mP(m)(a_m+i_m)\frac{\partial(x_k\pi)}{\partial y_k}+\frac{1}{2}\rho\beta\frac{k}{\langle k\rangle}s_k\sum_{m=1}^{n}mP(m)(a_m+i_m)\frac{\partial^2\pi}{\partial x_k^2}$$
$$-\frac{1}{2}\rho\beta\frac{k}{\langle k\rangle}s_k\sum_{m=1}^{n}mP(m)(a_m+i_m)\frac{\partial^2\pi}{\partial y_k^2}-\rho\beta\frac{k}{\langle k\rangle}s_k\sum_{m=1}^{n}mP(m)(a_m+i_m)\frac{\partial^2\pi}{\partial x_ky_k}+\rho\beta s_k\frac{k^2P(k)}{\langle k\rangle}(\frac{\partial(y_k\pi)}{\partial x_k}+\frac{\partial(z_k\pi)}{\partial x_k}),$$

Similar to the calculation above, we can get:

$$(\varepsilon_{S_k}^{+1}\varepsilon_{I_k}^{-1}-1)T(S_k-1,A_k,I_k+1|S_k,A_k,I_k)p(S_k,A_k,I_k,t)$$
$$=\sqrt{N_k}(1-\rho)\beta\frac{k}{\langle k\rangle}s_k\sum_{m=1}^{n}mP(m)(a_m+i_m)\frac{\partial\pi}{\partial x_k}+(1-\rho)\beta\frac{k}{\langle k\rangle}\sum_{m=1}^{n}mP(m)(a_m+i_m)\frac{\partial(x_k\pi)}{\partial x_k}$$
$$-\sqrt{N_k}(1-\rho)\beta\frac{k}{\langle k\rangle}s_k\sum_{m=1}^{n}mP(m)(a_m+i_m)\frac{\partial\pi}{\partial z_k}-(1-\rho)\beta s_k\frac{k^2P(k)}{\langle k\rangle}(\frac{\partial(y_k\pi)}{\partial z_k}+\frac{\partial(z_k\pi)}{\partial z_k})$$
$$-(1-\rho)\beta\frac{k}{\langle k\rangle}\sum_{m=1}^{n}mP(m)(a_m+i_m)\frac{\partial(x_k\pi)}{\partial z_k}+\frac{1}{2}(1-\rho)\beta\frac{k}{\langle k\rangle}s_k\sum_{m=1}^{n}mP(m)(a_m+i_m)\frac{\partial^2\pi}{\partial x_k^2}$$
$$-\frac{1}{2}(1-\rho)\beta\frac{k}{\langle k\rangle}s_k\sum_{m=1}^{n}mP(m)(a_m+i_m)\frac{\partial^2\pi}{\partial z_k^2}-(1-\rho)\beta\frac{k}{\langle k\rangle}s_k\sum_{m=1}^{n}mP(m)(a_m+i_m)\frac{\partial^2\pi}{\partial x_kz_k}$$
$$+(1-\rho)\beta s_k\frac{k^2P(k)}{\langle k\rangle}(\frac{\partial(y_k\pi)}{\partial x_k}+\frac{\partial(z_k\pi)}{\partial x_k}),$$

$$
\begin{aligned}
&(\varepsilon_{A_k}^{+1}-1)T(S_k,A_k-1,I_k|S_k,A_k,I_k)p(S_k,A_k,I_k,t)\\
=&\sqrt{N_k}\gamma_1 a_k\frac{\partial \pi}{\partial y_k}+\gamma_1\frac{\partial(y_k\pi)}{\partial y_k}+\frac{1}{2}\gamma_1 a_k\frac{\partial^2\pi}{\partial y_k^2},\\
&(\varepsilon_{I_k}^{+1}-1)T(S_k,A_k,I_k-1|S_k,A_k,I_k)p(S_k,A_k,I_k,t)\\
=&\sqrt{N_k}\gamma_2 i_k\frac{\partial \pi}{\partial z_k}+\gamma_2\frac{\partial(z_k\pi)}{\partial z_k}+\frac{1}{2}\gamma_2 i_k\frac{\partial^2\pi}{\partial z_k^2},\\
&(\varepsilon_{S_k}^{-1}-1)T(S_k+1,A_k,I_k|S_k,A_k,I_k)p(S_k,A_k,I_k,t)\\
=&-\sqrt{N_k}\alpha(1-s_k-a_k-i_k)\frac{\partial \pi}{\partial x_k}+\frac{1}{2}\alpha(1-s_k-a_k-i_k)\frac{\partial^2\pi}{\partial x_k^2}+\alpha(\frac{\partial(x_k\pi)}{\partial x_k}+\frac{\partial(y_k\pi)}{\partial x_k}+\frac{\partial(z_k\pi)}{\partial x_k}).
\end{aligned}
$$

Combing the equation of (13), (18) and making a comparison order by order, we can get the value of matrix A and B.

References

1. Dreessche, P. & Watmough, J. Reproduction numbers and sub-threshold endemic equilibria for compartmental models of disease transmission. *Math. Bio* **180**, 29–48 (2002).

2. Liu, J. & Zhang, T. Epidemic spreading of an seirs model in scale-free networks. *Communications in Nonlinear Science and Numerical Simulation* **16**, 3375–3384 (2011).

3. Thieme & Horst, R. Persistence under relaxed point-dissipativity (with application to an endemic model). *Siam Journal on Mathematical Analysis* **24**, 407–435 (2006).

4. Leenheer, P. & Smith, H. L. Virus dynamics: A global analysis. *Siam Journal on Applied Mathematics* **63**, 1313–1327 (2003).
